# Protective Effect of Procyanidin-Rich Grape Seed Extract against Gram-Negative Virulence Factors

**DOI:** 10.3390/antibiotics12111615

**Published:** 2023-11-10

**Authors:** Roberta Maria Nicolosi, Graziana Bonincontro, Elena Imperia, Camilla Badiali, Daniela De Vita, Fabio Sciubba, Laura Dugo, Michele Pier Luca Guarino, Annamaria Altomare, Giovanna Simonetti, Gabriella Pasqua

**Affiliations:** 1 Department of Environmental Biology, Sapienza University of Rome, P.le Aldo Moro 5, 00185 Rome, Italy; robertamaria.nicolosi@uniroma1.it (R.M.N.); graziana.bonincontro@gmail.com (G.B.); camilla.badiali@uniroma1.it (C.B.); daniela.devita@uniroma1.it (D.D.V.); fabio.sciubba@uniroma1.it (F.S.); gabriella.pasqua@uniroma1.it (G.P.); 2 Department of Science and Technology for Sustainable Development and One Health, University Campus Bio-Medico of Rome, Via Alvaro del Portillo 21, 00128 Rome, Italy; elena.imperia@unicampus.it (E.I.); l.dugo@unicampus.it (L.D.); 3 NMR-Based Metabolomics Laboratory (NMLab), Sapienza University of Rome, P.le Aldo Moro 5, 00185 Rome, Italy; 4 Research Unit of Gastroenterology, Department of Medicine and Surgery, University Campus Bio-Medico of Rome, Via Alvaro del Portillo 21, 00128 Rome, Italy; m.guarino@policlinicocampus.it; 5 Operative Research Unit of Gastroenterology, University Policlinico Foundation Campus Bio-Medico, Via Alvaro del Portillo 200, 00128 Rome, Italy

**Keywords:** grape seed extract, *Vitis vinifera* L., anti-virulence factors, LPS, bacterial biofilm, *Salmonella enterica* subsp. *enterica* serovar Typhimurium, *Escherichia coli*, *Galleria mellonella*, Caco-2

## Abstract

Biofilm formation and lipopolysaccharide (LPS) are implicated in the pathogenesis of gastrointestinal (GI) diseases caused by Gram-negative bacteria. Grape seeds, wine industry by-products, have antioxidant and antimicrobial activity. In the present study, the protective effect of procyanidin-rich grape seed extract (prGSE), from unfermented pomace of *Vitis vinifera* L. cv Bellone, on bacterial LPS-induced oxidative stress and epithelial barrier integrity damage has been studied in a model of Caco-2 cells. The prGSE was characterized at the molecular level using HPLC and NMR. The in vitro activity of prGSE against formation of biofilm of *Salmonella enterica* subsp. *enterica* serovar Typhimurium and *Escherichia coli* was investigated. In vivo, prGSE activity using infected *Galleria mellonella* larvae has been evaluated. The results show that the prGSE, if administered with LPS, can significantly reduce the LPS-induced permeability alteration. Moreover, the ability of the extract to prevent Reactive Oxygen Species (ROS) production induced by the LPS treatment of Caco-2 cells was demonstrated. prGSE inhibited the biofilm formation of *E. coli* and *S. Typhimurium.* In terms of in vivo activity, an increase in survival of infected *G. mellonella* larvae after treatment with prGSE was demonstrated. In conclusion, grape seed extracts could be used to reduce GI damage caused by bacterial endotoxin and biofilms of Gram-negative bacteria.

## 1. Introduction

In recent years, the association between increased intestinal permeability and oxidative stress production has been demonstrated in several chronic gastrointestinal (GI) disorders such as inflammatory bowel disease (IBD), bacterial infections, irritable bowel syndrome (IBS), and celiac disease [1,2,3].

The mechanisms of these pathological conditions are still unclear. However, the integrity of the intestinal barrier could be modulated by several factors—such as mucosal inflammatory events, different dietary patterns, and intestinal microbial composition [4]. Even if the connection between impaired intestinal permeability and the altered gut microbiota is still not clear, altered intestinal mucosal integrity is responsible for the translocation of the luminal content (bacterial products, pathogenic substances) to the bloodstream, as detected in chronic GI disorders [5,6]. The pathological passage of several substances can enhance the mucosal inflammatory processes based on chronic GI disorders, and, through the mesenteric lymph nodes, these pathogenic molecules can reach the systemic circulation contaminating sterile organs, including the liver, lungs, and brain [7].

One of the key mechanisms of pathological translocation involves lipopolysaccharide (LPS), an endotoxin found in Gram-negative bacteria like *Salmonella* species and *Escherichia coli*. These bacteria have been linked to various gastrointestinal diseases, including inflammatory bowel disease, irritable bowel syndrome, Crohn’s disease, and ulcerative colitis [8,9]. LPS, a component of the bacterial cell wall, plays a critical role in endotoxic shock and infection-related pathophysiology [10]. It can trigger both local and systemic immune/inflammatory responses, as seen in conditions like sepsis [7]. Endotoxemia has been identified in a significant proportion of patients with Crohn’s disease and ulcerative colitis [11]. Experiments have shown that the exposure of human colonic mucosa to pathogenic LPS can alter the contractility of colonic smooth muscle cells, suggesting that increased permeability may contribute to the development of gastrointestinal diseases [12,13,14,15]. Additionally, E. coli infection has been associated with reduced mucus thickness, compromised intestinal barrier integrity, and elevated LPS levels [9,11,16].

Pathogenesis in *Salmonella* spp. and *E. coli* infections is an outcome of multiple virulence factors, including biofilm production. Biofilm is a community of bacteria enclosed in an extracellular substance that renders bacteria resistant to stresses, immune system clearance, and antibiotics. Biofilm additionally mediates pathogen–host interactions and is an essential factor in chronic infections [17].

In the last years, in vitro and in vivo studies have demonstrated that several natural products could restore the altered intestinal permeability in stress conditions, suggesting the possibility of new therapeutic strategies [18,19,20,21]. Moreover, some of them could also modulate the oxidative stress processes and reduce pro-inflammatory cytokine secretion [3,4,8,22]

Phenolic compounds from plant matrices and phytochemicals showed different radical scavenging capacities [23]. Seed extracts of *Vitis vinifera* L. contain many bioactive molecules, lipids, proteins, carbohydrates, and 5–8% polyphenols, depending on the cultivar. Most of the total polyphenols are procyanidins (PCs), which are flavan-3-ols present in monomeric, dimeric, oligomeric, and polymeric forms. The PCs are considered to be primarily responsible for the biological effects of grape seed extract [24,25]. Annually, the agri-food supply chain generates large amounts of waste and by-products. Waste has no value and it cannot be reused within a production cycle, but must be disposed of. On the contrary, by-products as a secondary material can be reused even if they have less value than the primary material [23]. In particular, unfermented grape pomace is considered a by-product being used both for the production of grappa and as a fertilizer. It is obtained from the pressing of grapes, and it is mainly composed of seeds and skins, and a little amount of pulp [25]. Since it has been recognized as an important natural source of compounds with promising health properties, its use may be part of a circular economy perspective [25].

Several beneficial properties of grape products have been demonstrated, such as antiproliferative and cytotoxic effects in tumoral cell lines [9,26,27] and the inhibition of cellular cholesterol uptake and intestinal inflammatory processes [5,28,29,30].

Grape seed extract (GSE) rich in PCs has shown higher antioxidant activity than other plant extracts [31]. Moreover, it has been reported that GSE, obtained from different table and wine cultivars, has a significant activity against fungi such as *Candida* species, dermatophytes, and *Malassezia* spp. [24,25]. Kitsiou and colleagues demonstrated a significant inhibitory growth effect of 4% GSE against *Salmonella enterica* subsp. *enterica* serovar Typhimurium and *E. coli* [32]. GSE, containing high levels of PCs rich in phenolic hydrogen scavengers of hydrogen radical donors, could reduce oxidative stress by acting as a regulator of the inflammatory reaction, restoring the integrity of the intestinal barrier after LPS exposition [33]. In an interesting previous study, it has been demonstrated that poorly bioavailable grape polyphenols can contrast the pro-inflammatory action of LPS in a model of Caco2 cells (Human epithelial colorectal adenocarcinoma cell line), possibly preventing damage to intestinal mucosal permeability [28]. In summary, grape polyphenols have previously been shown to improve gut health, preventing oxidative damage; however, the knowledge about these anti-inflammatory effects is still not extensive.

For these reasons, in the present study, the effect of procyanidin-rich GSE (prGSE), obtained from a wine vine farm of the Lazio Region in Italy, has been studied on bacterial LPS-induced oxidative stress and epithelial barrier integrity damages in a model of Caco-2 cells. Among two of the major foodborne Gram-negative pathogens, *S.* Typhimurium and *E. coli*, the efficacy of prGSE inhibiting the formation of biofilms was evaluated. Moreover, the protective effect of prGSE in infected *Galleria mellonella* larvae, a consolidated in vivo model, has been investigated for the first time.

## 2. Results

### 2.1. Phytochemical Analysis of prGSE

#### 2.1.1. Total Phenolic Content

The results of chemical analyses of *V. vinifera* L. (cv Bellone) seed extract from unfermented pomace reveal that it is a very phenol-rich matrix. The spectrophotometric analysis and subsequent quantification based on the calibration curve of gallic acid have been provided to measure the total phenolic compounds at a concentration of 645 mg/g.

#### 2.1.2. High-Performance Liquid Chromatography (HPLC) Analysis

Separation of phenolic compounds in GSE was achieved within 90 min. The HPLC chromatogram of GSE was recorded at 278 nm. The components were eluted in the following order: catechin (1), procyanidin B2 (2), epicatechin (3), epicatechin gallate (4) and the polymeric procyanidins (5 and 6); their concentrations are shown in Table 1. Moreover, two groups of polymeric procyanidins, named Pol 1 (5) and Pol 2 (6), have been separated and quantified. As shown in Table 1, the main components of GSE are 5 and 6, with concentrations of 365.48 and 78.36 mg/g, respectively. Within the monomeric procyanidins, catechin (1) represents the main one with a concentration of 10.92 mg/g, followed by epicatechin (3) (7.66 mg/g) and epicatechin gallate (4) (2.80 mg/g). Finally, procyanidin B2 (2) shows a concentration of 4.05 mg/g. The total procyanidins content determined by HPLC-DAD is 469.27 mg/g (Table 1).

#### 2.1.3. Nuclear Magnetic Resonance (NMR) Analysis

**Figure 1:**
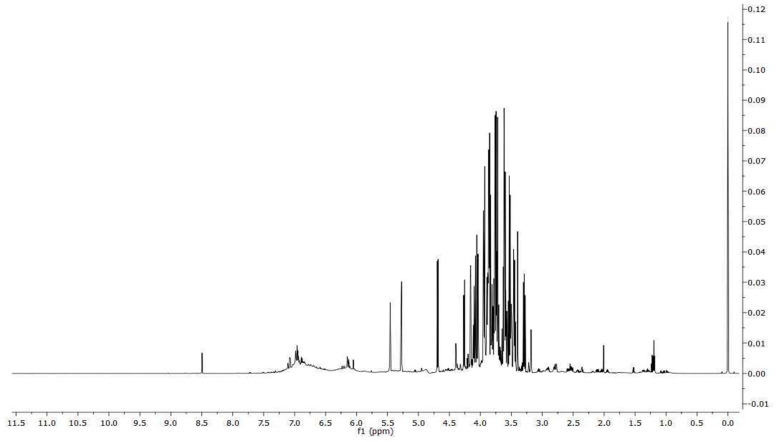
^1^H spectrum of *V. vinifera* seed extract.

From the ^1^H NMR spectrum (Figure 1), it is possible to identify and quantify 24 metabolites classified as amino acids, organic acids, carbohydrates and miscellaneous molecules. Among them, we might note the presence of ascorbate, procyanidin B1 and polymeric procyanidins. In particular, the diagnostic resonances of procyanidin B1 are the doublets at 6.05 ppm, 6.13 ppm, 6.15 ppm and two ABX systems at 6.88 ppm (two doublet of doublets), 6.95 ppm (triplet), 6.96 (triplet), 6.98 ppm (doublet) and 7.06 ppm (doublet). As regards the aromatic moieties of the polymeric procyanidins, they can be assessed by the broad resonances at 6.15 ppm and 6.82 ppm. The carbohydrates, whose resonances are the most abundant in the spectrum, are identified on the basis of their anomeric protons at 4.65 ppm and 5.22 ppm for glucose and at 5.44 ppm for sucrose. The identified molecules were integrated and normalized for the number of protons originating that resonance, then by the internal standard, and then for the extract dry weight. The measured amounts are reported in Table 2.

### 2.2. Caco-2 Experiments

#### 2.2.1. Effect of the Different Treatments with LPS, prGSE, and LPS-prGSE on the Caco-2 Cell Monolayer Viability

The effects of prGSE on cell viability using the MTT assay have been preliminarily assessed. To this end, Caco-2 cells were exposed to increasing concentrations (3.125–6.25–12.5–25 and 50 µg/mL) of prGSE for 24, 48 and 72 h. The MTT assay results suggest that prGSE applied at 3.125–6.25–12.5 µg/mL had no significant cytotoxic effects on the Caco-2 cells after treatment (96.8 ± 3.04 vs. 100 ± 0.54%; 92.8 ± 2.08 vs. 100 ± 0.54%; 84.7 ± 1.056 vs. 100 ± 0.54% respectively, n.s.) compared to control (untreated cells). The concentrations of 25 and 50 µg/mL had a significant cytotoxic effect on the Caco-2 cells compared to control (untreated cells) (77.47 ± 4.098 vs. 100 ± 0.54%; 76.29 ± 2.009 vs. 100 ± 0.54% respectively, *p* < 0.05). In the following experiments, a prGSE concentration of 6.25 µg/mL was used because, based on preliminary experiments, it turned out to be the minimum effective dose in obtaining the described results. Moreover, in a previous paper, the dose of 12.5 of another GSE has already shown its ability to prevent oxidative stress and permeability damage due to LPS in Caco-2 cells [28].

#### 2.2.2. FD-4 Permeability Analysis after LPS, prGSE, and LPS-prGSE on the Caco-2 Cells

**Figure 2:**
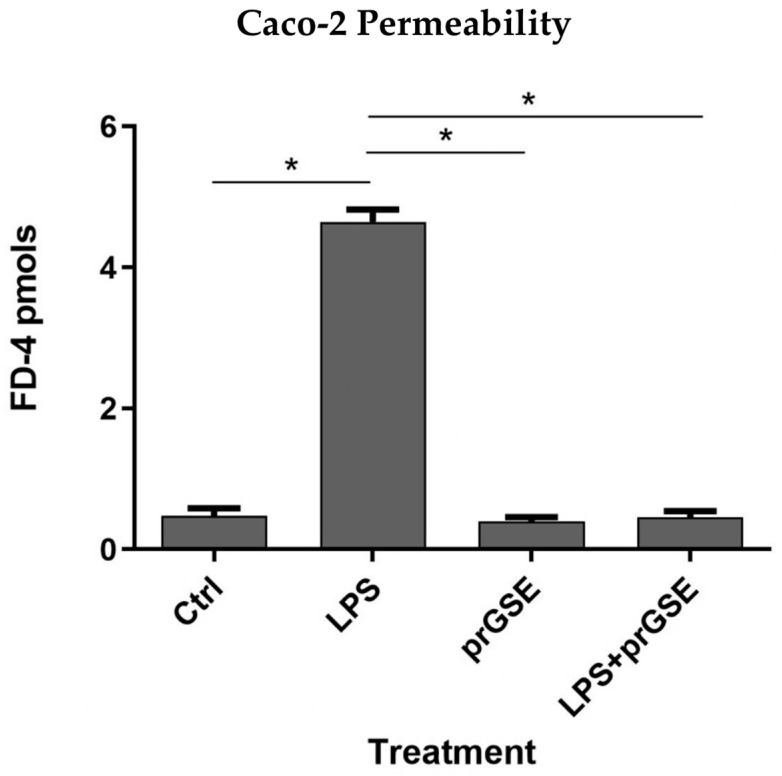
The barrier function of the Caco-2 cell monolayers was evaluated through the FD-4 (fluorescein isothiocyanate-dextran) permeability assay. LPS treatment (10 µg/mL) for 24 h, prGSE (6.25 µg/mL) for 24 h and LPS with prGSE treatment for 24 h. The experiments were repeated in triplicate. Data are reported as mean ± SD. Statistical analysis was performed using a two-way ANOVA, followed by Bonferroni’s post-hoc correction test. * *p* < 0.05. Ctrl (control); LPS (lipopolysaccharide); prGSE (procyanidins-rich grape seed extract); prGSE+LPS (mix).

To evaluate the alteration of the Caco-2 cell’s monolayer integrity, the paracellular passage of FD-4 (with a molecular weight of 4 kDa) across Caco-2 monolayers was evaluated using a multi-plate reader (Tecan Italia srl, Milan, Italy), and was calculated and plotted as a function of time every 20 min over 6 h (from 20 min up to 360 min in Figure 1). FD-4 permeability across the epithelial barrier is significantly increased following LPS treatment (10 μg/mL for 24 h) compared to the control (4.647 ± 0.306 vs. 0.479 ± 0.178 pmols; *p* < 0.0313), which agrees with previous studies [34,35,36,37]. Co-treatment with prGSE was able to prevent the LPS-induced alteration (4.647 ± 0.306 vs. 0.457 ± 0.147 pmols; *p* < 0.0313), showing a value similar to the control. Interestingly, prGSE alone did not alter the permeability values (0.397 ± 0.103 vs. 0.479 ± 0.178 pmols, *p* = ns), excluding a possible harmful effect of the extract (Figure 2).

#### 2.2.3. Analysis of Reactive Oxygen Species (ROS) Production after LPS, prGSE, and LPS-prGSE Treatment on the Caco-2 Cells

**Figure 3:**
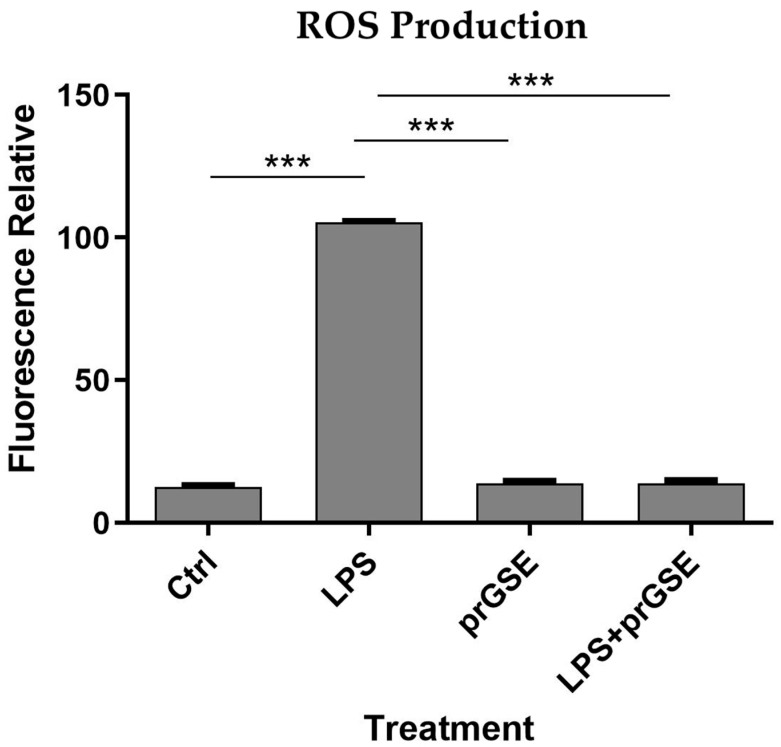
Reactive Oxygen Species (ROS) analyses were performed using carboxy-H2DCFDA staining by a fluorescence plate reader. The experiments were executed in triplicate. Data are reported as fluorescence relatives. The experiments were executed in triplicate. Data are reported as mean ± SD. Statistical analysis was performed using a two-way ANOVA, followed by Bonferroni’s post-hoc correction test *** *p* < 0.0001. Ctrl (control); LPS (lipopolysaccharide); prGSE (procyanidins-rich grape seed extract); prGSE+LPS (mix).

In order to explore the presence of oxidative stress following exposure to the different treatments analyzed, intracellular ROS levels were measured in LPS-, prGSE-, and LPS+prGSE-treated Caco-2 monolayers via the carboxy-H2 DCFDA fluorescent probe, as described previously [38,39]. LPS treatment significantly increased the ROS production compared to the control (104 ± 1.90 vs. 12.9 ± 1.08; *p* < 0.0001) while the co-treatment prGSE-LPS, with the prGSE concentration of 6.25 µg/mL, prevented this production (19.0 ± 2.00 vs. 104 ± 1.90; *p* < 0.0001). prGSE alone did not determine a significant production of ROS compared to the control (11.3 ± 1.30 vs. 19.2 ± 2.0; *p* > 0.0001). Data are reported in Figure 3.

### 2.3. In Vitro and In Vivo Activity of prGSE against S. Typhimurium and E. coli Cells and Virulence Factors

#### 2.3.1. In Vitro Antibacterial Activity Evaluation

Using the standard microdilution method, the PrGSE antibacterial activity was investigated against *S.* Typhimurium ATCC 14028 and *E. coli* ATCC 25922 [40]. PrGSE showed the capacity to inhibit Gram-negative bacteria growth in a dose-dependent manner, being more active against *S.* Typhimurium. MIC values for *S.* Typhimurium are lower than those obtained with *E. coli*. PrGSE displayed a Geometric Mean (GM) MIC_50_ value of 44.17 µg/mL against *S.* Typhimurium, while for *E. coli* the GM MIC_50_ value is 55.58 µg/mL (Table 3).

#### 2.3.2. In Vitro Antibiofilm Activity

**Figure 4:**
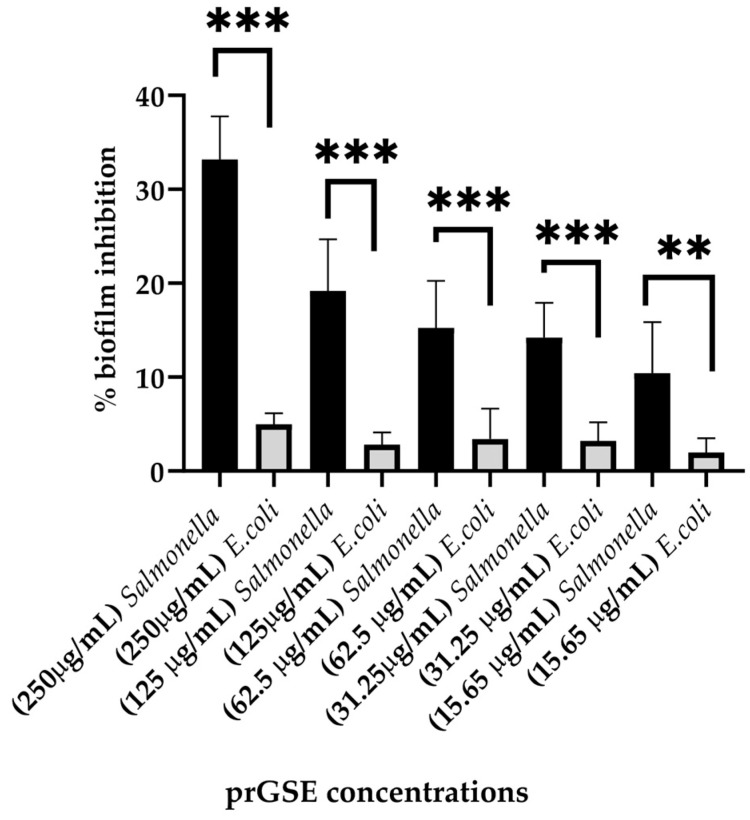
Percentage of inhibition biofilm formation of *E. coli* ATCC 25922 and *S.* Typhimurium ATCC 14028 with scalar concentrations of procyanidins-rich grape seed extract (prGSE) (250 µg/mL, 125µg/mL, 62.5 µg/mL, 31.25 µg/mL and 15.65 µg/mL) after 24 h of incubation. ** *p* < 0.01; *** *p* < 0.001.

The anti-biofilm activity was evaluated after 24 h of incubation using the CV assay. prGSE displayed a major anti-biofilm activity against *S.* Typhimurium, with a percentage of inhibition of 33% at 250 µg/mL. At the same concentration, prGSE inhibits *E. coli* biofilm formation by nearly 5%. Thus, it can be stated that prGSE has no activity against *E. coli* biofilm. On the other hand, prGSE showed a dose-dependent inhibition of *S.* Typhimurium biofilm formation. As shown in Figure 4, the percent rate of inhibition decreases with the concentration of prGSE used.

#### 2.3.3. In Vivo Antibacterial Activity

**Figure 5:**
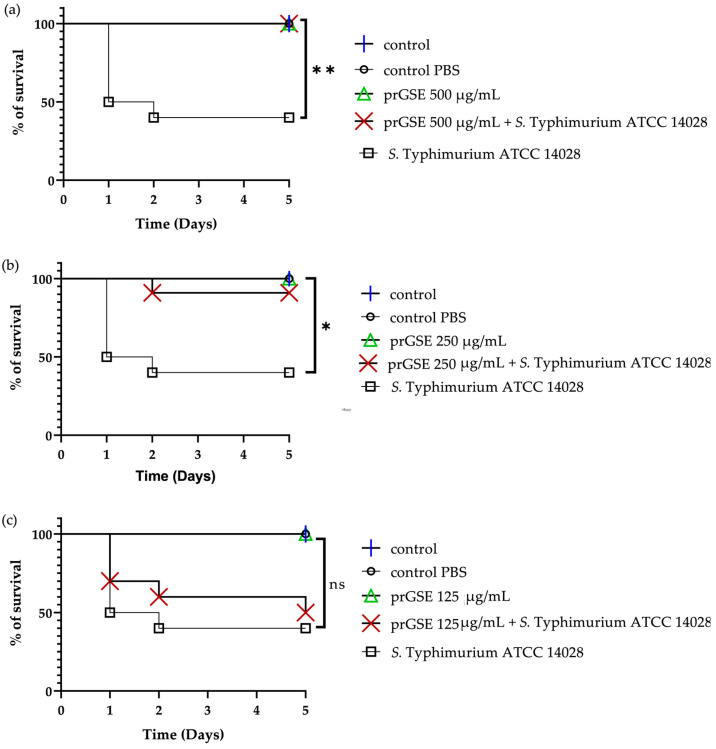
Survival curves of *G. mellonella* larvae infected with *S.* Typhimurium ATCC 14028 and treated with 500 µg/mL (**a**), 250 µg/mL (**b**), and 125 µg/mL (**c**) of procyanidins-rich grape seed extract (prGSE). PBS = Phosphate-Buffered Saline. The Mantel–Cox log-rank test was used to judge the statistical significance relative to the control. * *p* < 0.05 compared to the control; ** *p* < 0.01 compared to the control; ns = not significant.

**Figure 6:**
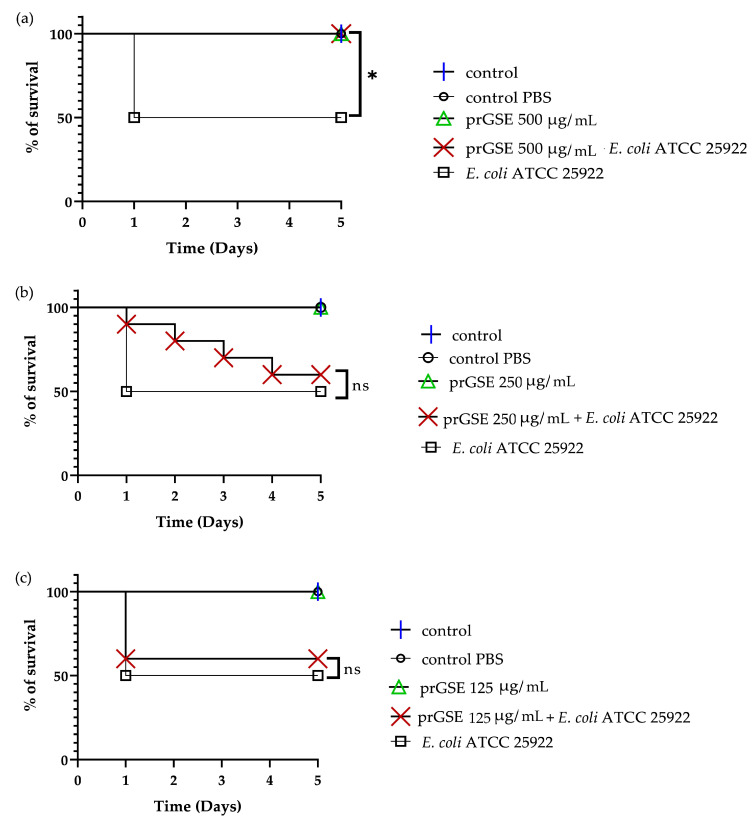
Survival curves of *G. mellonella* larvae infected with *E. coli* ATCC 25922 and treated with 500 µg/mL (**a**), 250 µg/mL (**b**) and 125 µg/mL (**c**) of procyanidins-rich grape seed extract (prGSE). PBS = Phosphate Buffered Saline. The Mantel–Cox log-rank test was used to judge the statistical significance relative to the control. * *p* < 0.05 compared to the control; ns = not significant.

In the past decades, *G. mellonella* larvae have been largely used to test the antimicrobial activity of both chemical and natural compounds because their immune system is remarkably similar to that of mammals. This animal model meets the bioethics principle of the 3Rs (Replacement, Reduction, and Refinement) in animal experimentation and is characterized by simple handling and experimental procedures [41]. *G. mellonella* larvae were infected with *S.* Typhimurium ATCC 14028 and *E. coli* ATCC 25922. After they were infected, they were treated with 10 µL of 500 µg/mL, 250 µg/mL, or 125 µg/mL of prGSE. The survival of the larvae was reported daily for 5 days. The survival rate was 100% for both untreated larvae and larvae injected with PBS. Conversely, the mortality rate in the group infected with *S.* Typhimurium and *E. coli* was 60% and 40%, respectively. The in vivo antibacterial assay showed significant results at the concentrations of 500 µg/mL and 250 µg/mL (*p* value < 0.01 and 0.05, respectively) against *S.* Typhimurium (Figure 5). When treated with 5 µg/larva of prGSE, *G. mellonella* larvae infected with *S.* Typhimurium showed a survival rate of 100%. After 5 days, only one larva treated with 5 µg/larva of prGSE died. The survival rate decreased to 50% after 5 days for the group of larvae treated with 2.5 µg/larva of prGSE. *E. coli*-infected larvae, treated with 5 µg/larva of prGSE, survived with a percent rate of 100%; 40% of larvae died in the group treated with 2.5 µg/larva and in that treated with 1.25 µg/larva of prGSE after 5 days (Figure 6).

## 3. Discussion

The recovery of *V. vinifera* seeds from unfermented pomace aligns with the principles of the circular economy; this model of production and consumption can exploit existing products and natural resources with the aim of extending their life cycle and reducing by-products and waste derived from them. This study shows how *V. vinifera* seeds from unfermented pomace can be reintroduced into production cycles due to their containing several bioactive compounds. The phytochemical characterization of prGSE obtained from the cultivar Bellone showed the presence of several phenolic compounds. From a qualitative point of view, the prGSE of the present investigation shows the same phytochemical profile as the other cultivars, with as its main components monomeric, dimeric, and polymeric procyanidins [24].

PrGSE has a high concentration of polymeric procyanidins (443.84 mg/g over 469.27 mg/g of total procyanidins, as measured by HPLC), and these results could explain the high activity of this extract. In fact, in a previous study, a significant correlation between the content of polymeric flavan-3-ols with a polymerization degree ≥ 4 in GSEs and antimicrobial activity was demonstrated [24].

In this study, the effects of prGSE on the damages caused by LPS and on the inhibition of the biofilm formation of two Gram-negative bacteria, *E. coli* and *S.* Typhimurium, have been evaluated. Furthermore, the antibacterial activity of prGSE was evaluated in a *G. mellonella* in vivo model.

The effect of LPS on intestinal mucosa is not entirely understood; it is already known that it can alter intestinal permeability [42], possibly facilitating a pathological translocation of several substances. Moreover, it has been demonstrated that it binds the Toll-Like Receptor 4 on the surface of the intestinal epithelial cells, activating several local inflammatory processes, such as ROS production and the synthesis of inflammatory cytokines [43,44].

The present study investigated the effect of prGSE against LPS-induced damage on Caco-2 cells. Caco-2 was chosen for this test since it is a model of the human epithelial colorectal adenocarcinoma cell line that could be considered as an intestinal epithelium model because it forms monolayers with characteristics of intestinal epithelial cells, such as the formation of microvillus, and it expresses brush-border proteins [12,45].

The results of the present investigation confirm that prGSE, if administered with LPS, significantly reduces the permeability alteration due to this endotoxin. It has been previously demonstrated, in an animal model of intestinal inflammation and in cell cultures, that PCs are able to prevent impaired intestinal permeability even if it seems that the polymeric PCs mediate this protective effect but not the oligomers [28,46,47]. In accordance with this evidence, the prGSE was rich in polymeric procyanidins. The positive effect of PCs on the altered permeability induced by LPS seems to be induced by the overexpression of tight junction proteins such as occludin and zona occludens (ZO)-1 [48,49]. Claudin-2 is a pore-forming claudin that forms high conductance, paracellular cation-selective pores [50], and it has been demonstrated that in IBD patients, its expression is altered, determining a change in tight-junction structure [51,52].

Another important result of the present investigation was the ability of the extract to prevent ROS production induced by the LPS treatment of Caco-2 cells, confirming previous evidence in which a GSE significantly decreased the LPS-induced production of intracellular reactive oxygen species (ROS) and mitochondrial superoxide in a Caco-2 model [28]. It is well known that oxidative stress is one of the most critical mechanisms of the inflammatory processes, due to Gram-negative infection in the intestinal mucosa [53,54,55].

For several years, the antioxidant and anti-inflammatory capacities of proanthocyanidin-rich plant products have been explored with a view to identifying new potential strategies for preventing and/or treating acute and chronic intestinal disorders [56]. For this reason, in this study, the protective role of prGSE was explored to identify new natural substances derived from by-products capable of being reused for preventive and curative purposes. It was also interesting to observe that the treatment of Caco-2 with the extract alone did not alter the mucosal permeability, nor did it determine the production of ROS, confirming that this extract does not cause alterations to the intestinal epithelial cells [3].

Two Gram-negative bacterial species frequently involved in acute and chronic intestinal diseases are *E. coli* and *S.* Typhimurium, which affect millions of people yearly [57]. *Salmonella* spp. could be responsible for chronic intestinal infection, gut microbiota alteration, and the genesis of inflammatory processes [58]. The present study demonstrated that prGSE inhibits the formation of *S*. Typhimurium biofilm, which is responsible for drug resistance and intestinal damage. The formation of biofilm is essential in chronic human intestinal infections because it allows *S*. Typhimurium to persist in the inflammation environment, accelerate mucosa damage, and creates an immune cell-related chronic infection reservoir [5,9,59]. The results showed a significantly higher effect of the extract on *S.* Typhimurium biofilm than on *E.coli.* The reason for this difference in activity will be further investigated. Hypotheses can be made on the basis of previous studies on the activity of molecules in GSE on *S*. Typhimurium. *S*. Typhimurium biofilm-bound proteins are under the control of quorum sensing (QS) pathways. Hosseinzadeh and colleagues reported that epigallocatechin inhibited the expression of QS-associated genes, such as *luxS* of *S*. Typhimurium [60]. Surette and colleagues demonstrated that domesticated laboratory strains of *E. coli* do not produce the LuxS protein. The LuxS protein, other than being important in biofilm formation, helps regulate the transition from commensal to pathogenic [61]. In addition to the difference shown in in vitro activity, another element that could support this hypothesis is the lower mortality of *G. mellonella* larvae found after infection with *E. coli* compared to *S.* Typhimurium.

For the first time, in this study, the activity of prGSE in *G. mellonella* larvae infected with *E. coli* and *S.* Typhimurium has been investigated. The *G. mellonella* in vivo model can be considered as an alternative to vertebrates to investigate enteric bacteria pathogens [62], since similarities have been described between the mammalian digestive apparatus and intestinal epithelial cells from larvae [63]. The primary tissue architecture and some microbial communities of the *G. mellonella* midgut are similar to those found in the human intestine [62].

Lately, a reduction in *Listeria monocytogenes* virulence and *Staphylococcus aureus* infections has been successfully demonstrated using *G. mellonella* larvae [64,65,66]. Additionally, *G. mellonella* has been used to evaluate the infectivity of gut pathogens such as *L. monocytogenes* [67], *Campylobacter jejunis* [68], *Vibrio* spp. [69], *Shigella* spp. [70], and *S. enterica* [71].

The activity of prGSE has been demonstrated in the *G. mellonella* infection model. The results obtained in the *G. mellonella* infection model show the activity of prGSE by reducing the mortality of both the larvae infected with *S.* Typhimurium and the larvae infected with *E. coli*. The highest dose used in the present study was 16.7 mg/kg of larval weight. This dose significantly reduces the mortality of larvae infected with both *S.* Typhimurium and *E. coli.* This dose, which is effective for fighting the infection in the larva, can be compared to the effective dose in humans. As reported by some authors, many studies have used clinically relevant doses of antibiotics for human infection. The authors demonstrated that this model is useful for approximating the antibiotic response in humans [72,73].

The protective effects exerted in the *G. mellonella* larvae could be due to several mechanisms, including the increase in the larval immune system [74].

It is known that pathogenicity, virulence factors, biofilm formation, and antioxidant activity are linked. When ROS accumulates within the cells, oxidative stress occurs. Oxidative stress plays an important role in biofilm formation. ROS can act to promote microbial attachment, consequently leading to biofilm development [75]. The hypothesis of the present investigation is that the inhibition of biofilm formation is related to the antioxidant action of the extract, and that this increases the survival of G. *mellonella*. Further investigations are needed to explore this potential mechanism.

In the present study, the results have demonstrated that prGSE can modulate some important virulence factors of Gram-negative bacteria, suggesting a possible use of this extract as an alternative treatment for maintaining gastrointestinal health.

A limitation of this study is that it primarily focuses on the effects of grape seed extract on *S.* Typhimurium and *E. coli.* It does not address the potential impact on the intestinal microbiota. Further research using the human intestinal environment may be necessary to confirm the applicability of these findings in clinical contexts. Moreover, further investigations, based on these preliminary results, are necessary to deeper explore the mechanisms responsible for GSE beneficial effects.

In conclusion, further studies are needed to confirm these protective and anti-inflammatory properties, even if these preliminary results are in line with the current literature and are promising in terms of possible uses in clinical practice.

## 4. Materials and Methods

### 4.1. Extraction and Phytochemical Analysis of GSE

#### 4.1.1. Plant Material

*Vitis vinifera* L. cv Bellone (IVD-var_356) was cultivated in the Pietra Pinta winery factory, located in Cori (Latina, Latium region, geographical coordinates 41°64′37″ N, 12°88′71″ E). The botanical species was identified by Prof. Daniela De Vita and Prof. Gabriella Pasqua through morphological comparison with data available in the literature [76]. Plant material was harvested in September 2021 and the pomace obtained by pressing was immediately stored at −20 °C until use. A sample of this pomace is kept in our laboratory under the voucher code 20210901.

#### 4.1.2. Sample Preparation

The seeds were separated from the unfermented pomace by sieving, washed and frozen to facilitate the freeze-drying process. The freeze-dried seeds were weighed and ground into a fine powder. They were extracted with an EtOH/H_2_O mixture (7:3 *v*/*v*) acidified with formic acid until pH 3, using the matrix/solvent ratio of 1 g/10 mL. Extraction was conducted at 40 °C for 3 h. The removal of the solid residue was achieved by vacuum filtration; the filtrate was then concentrated using the rotary evaporator (BUCHI R Pro 220, BUCHI Italia s.r.l, Cornaredo, Italy). Finally, the extract was frozen and lyophilized.

#### 4.1.3. Total Phenolic Content

For the total phenolic compounds’ quantification, the Folin–Ciocalteu method was performed. A stock solution of gallic acid with a concentration of 2.4 mg/mL and different dilutions were prepared for the calibration curve. A stock solution of gallic acid with a concentration of 2.4 mg/mL and different dilutions were prepared for the calibration curve (y = 5.4072x + 0.0211; R^2^ = 0.9919). A 50% EtOH/H_2_O solution of the lyophilized extract with a concentration of 1 mg/mL and a sodium carbonate solution at 10.75% were prepared. At room temperature, 1 mL of sample solution was placed in a 20 mL volumetric flask, and 2 mL of distilled water and 1 mL of Folin–Ciocalteu reagent were then added to the flask. Following the addition of Folin–Ciocalteu reagent, a waiting time of 3 min was observed. Afterward, 4 mL of sodium carbonate solution and 12 mL of distilled water were added to the flask. A blank with all the components except the extract was also prepared. The samples were then shaken and incubated at room temperature for 90 min. Subsequently, the samples were placed in a quartz cuvette cell and then read on a spectrophotometer (Shimadzu UV-1280, Shimadzu Italia S.r.l., Milan, Italy) at an absorbance of 750 nm [77]. All total phenolic compound concentrations have been expressed in gallic acid equivalents.

#### 4.1.4. High-Performance Liquid Chromatography (HPLC) Analysis

HPLC analyses were performed with an HPLC system consisting of a 1260 Infinity II flexible pump, a 1260 Infinity II autosampler, and an HS 1260 Infinity II diode array detector set at lambda of 278 nm (Agilent, Santa Clara, CA, USA). An InfinityLab Poroshell 120 EC-C18 column (3.0 mm × 150 mm, 2.7 µm) (Agilent, Santa Clara, CA, USA) was used as a stationary phase at a temperature of 27 °C. The gradient elution method was performed starting with 95% H_2_O for 5 min, then with 84% H_2_O for 2 min, 80% H_2_O for 4 min, 70% H_2_O for 3 min, 20% H_2_O for 4 min, until reaching 100% ACN for 5 min, followed by an equilibration time of 10 min with a flow rate of 1 mL/min. A stock solution of the standards (catechin, procyanidin B2, epicatechin and epicatechin gallate) was prepared each at the concentration of 1 mg/mL except for procyanidin B2, the concentration of which was 0.5 mg/mL, and this was solubilized in a 70% (*v*/*v*) hydroalcoholic mixture. Five dilutions were performed from the stock solution and a volume of 2 µL was injected to construct calibration curves for each standard, and elution times and peak subtended areas were identified. The seed extract at the concentration of 10 mg/mL was solubilized in the same 70% hydroalcoholic mixture and 5 µL was injected for a total of three replicates. Polymeric procyanidins with a degree of polymerization ≥4 were quantified at 278 nm according to the method described by Simonetti and colleagues [24] using procyanidin B2 as an external standard in a concentration range of 0.001–0.2 μg and a five-point calibration curve with R^2^ = 0.9998.

#### 4.1.5. Nuclear Magnetic Resonance (NMR) Analysis

A known amount of dried extract of *V. vinifera* seeds was resuspended in 700 μL of D_2_O containing 3-(trimethylsilyl)-propionic-2,2,3,3-d_4_ acid sodium salt (TSP) as an internal chemical shift and concentration internal standard at a final concentration of 2 mM. The NMR experiments were carried out at 298 K on a JNM-ECZ 600R (JEOL Ltd., Tokyo, Japan) spectrometer operating at the proton frequency of 600 MHz and equipped with a multinuclear z-gradient inverse probe head, and the monodimensional ^1^H spectra were acquired employing a *presat* pulse sequence, 64 k data points, a spectral width of 15 ppm (9.03 kHz), a recycle delay of 5.72 s, a *presat* time of 2 s and an acquisition time of 5.81 s. The spectra were processed with an exponential window function of 0.1 Hz and phase-corrected, and then the baseline was optimized with the Akima algorithm. Resonance assignment was carried out on the basis of resonance chemical shift, multiplicity, software database (Chenomx 9.0, Chenomx Inc., Edmonton AB, Canada) and literature data [78,79].

### 4.2. Caco-2 Culture Experiments

#### 4.2.1. Cell Culture

Human epithelial colorectal adenocarcinoma (Caco-2) cells were acquired from the American Type Culture Collection (ATCC, Manassas, VA, USA) and were cultured following standard procedures. The cells were grown in a humidified incubator at 37 °C with 5% CO2 in Dulbecco’s modified eagle’s medium (DMEM) containing 4.5 g/L of glucose and L-glutamine, without sodium pyruvate (DMEM high glucose, Corning, Sigma-Aldrich, Milan, Italy) containing 10% (vol/vol) fetal bovine serum (FBS, Euroclone, S.P.A, Milan, Italy). After thawing the cells, FBS was added at 20%, then the Caco-2 cell line was maintained using DMEM with 10% FBS-5% (vol/vol) L-glutamine (Aurogene, Rome, Italy), 5% (vol/vol) Penicillin/Streptomycin (Merck, Sigma-Aldrich, Milan, Italy) and 0.5% (vol/vol) HEPES (Dominique Dutscher, Issy-les-Moulineaux, France). L-glutamine 2 mM was added after 21 days to avoid its degradation. Caco-2 cells were periodically checked to exclude contamination. Cells seeded at a density of 5.0 × 10^4^ per insert were grown on transwell chambers (12 mm with 0.4 μm pore polyester membrane inserts; Corning, Sigma-Aldrich, Milan, Italy) placed in a 12-well plate: 500 μL of media were placed in the apical compartments and 1600 μL of media were placed in the basolateral compartments. Experiments were performed 21 days after seeding when the cells reached confluence and differentiation. The media was changed every other day in the apical and basolateral compartments of the well until the day of experimentation [12]. The experiments were conducted 21 days after seeding, once the cells had achieved confluence and differentiation. Throughout this period, fresh media was replenished every other day in both the apical and basolateral compartments of the well, following the protocol described in [12].

#### 4.2.2. Caco-2 Cell Monolayer Viability Assay

To determine the effects of extracts on Caco-2 cells and to choose the correct concentration to use for experiments, viability was assessed using an MTT assay after 24, 48 and 72 h of exposure of prGSE extract at different concentrations (from 3.125 to 50 µg/mL), as previously described [28]. The concentrations examined in Caco-2 cells were chosen based on previous studies, in which it has been demonstrated that doses higher than 50 ug/mL have antiproliferative and cytotoxic effects in this human epithelial colorectal adenocarcinoma cell line [26,27].

Subsequently, the culture medium was aspirated, and each well was rinsed with 200 mL of phosphate-buffered saline (PBS) (Sigma-Aldrich, Milan, Italy). The assessment of cellular metabolic activity was carried out by measuring their ability to convert the yellow MTT (0.5 mg/mL in serum-free DMEM) into a blue formazan product during a 3 h incubation period at 37 °C. Then, the medium containing MTT was removed from each well of the 96-multiwell and washed in PBS, and the formazan crystals were dissolved in DMSO (Sigma-Aldrich, Milan, Italy). The absorbance was read at 570 nm using a microplate reader (Tecan Infinite M200-Pro, Tecan, Milan, Italy). Cell proliferation values were expressed as percentages from the relative absorbance measured in the treated wells versus control wells (untreated cells). Cell proliferation values were quantified as percentages, with reference to the absorbance measured in the treated wells in comparison to the control wells, represented by untreated cells. Each test was performed in triplicate.

#### 4.2.3. Treatment of Caco-2 Cells with LPS and prGSE

Cells were challenged apically with LPS, prGSE, and LPS + prGSE for 24 h at 37 °C. LPS (bacterial lipopolysaccharide from a pathogenic strain of *E. coli* 0111:B4 *w*/*v*, Sigma-Aldrich, Milan, Italy) at the concentration of 10 mg/mL [12] and prGSE (6.25 mg/mL) were dissolved in plain DMEM. LPS was used as a positive control, as the literature has shown that it reduces cell viability [12].

#### 4.2.4. Cell Permeability Assay

Epithelial barrier integrity was assessed by quantifying the unidirectional paracellular transport of fluorescein isothiocyanate-dextran (FD-4, Sigma-Aldrich, Milan, Italy) from the apical to basolateral compartments, following established protocols [12]. In brief, before starting transport studies, the culture medium was aspirated from both sides of the Caco-2 cell monolayer, and replaced with prewarmed Krebs-Hensleit Buffer with 2000 mg/L glucose, without calcium chloride and sodium bicarbonate (KHB, pH 7.4 Sigma-Aldrich, Milan, Italy) for 1 h at 37 °C in the dark. The cells were subsequently rinsed with PBS to remove red-phenol dye, and FD-4 was added to the apical side of the Caco-2 monolayer at a final concentration of 1 mg/mL in prewarmed KHB [12]. Paracellular permeability was calculated from apical to basolateral transport, with undernatants being collected. The concentration of FD-4 in the solution was measured every 20 min over a 6 h period by withdrawing an aliquot of 200 µL from the basolateral compartment (undernatants) and replacing it with an equal volume (200 µL) of fresh KHB. Fluorescence readings were taken at excitation/emission wavelengths of 490/520 nm using a multi-plate reader Tecan (Tecan Italia srl, Milan, Italy). Fluorescence values were converted into fluorescein concentration (pmol) based on a standard curve generated from different concentrations of the FD-4 probe. Each experiment was performed in triplicate, with independent control groups for all three conditions. Untreated cells served as the control group.

#### 4.2.5. Analysis of Oxidative Stress

Intracellular ROS production was assessed by adding 2′,7′-dichlorodihydrofluorescein diacetate (H2-DCF-DA, Sigma Aldrich, Milan, Italy), as previously described [12], with slight modifications in Caco-2 cells exposed to LPS, prGSE and LPS + prGSE for 24 h in complete medium. The day of the experimentation, cells were incubated for three hours. Incubation with 400 μM H_2_O_2_ (Sigma-Aldrich, Milan, Italy) was used as a positive control for ROS. After incubation, the culture medium was replaced with PBS and cells were loaded with 10 µM H2-DCF-DA for 30 min. After incubation, PBS and H2-DCF-DA were removed and the cells were gently washed twice in PBS. Then, a red-free medium was added. The increase in cell fluorescence was measured at excitation and emission wavelengths of 485 and 530 nm, respectively, using an Infinite F200 auto microplate reader (Tecan Italia srl, Milan, Italy) at 25 °C. Each experiment was performed in triplicate with independent controls among the three conditions. The untreated cells were used as controls.

### 4.3. In Vitro and In Vivo Activity of prGSE against S. Typhimurium and E. coli Cells and Virulence Factors

#### 4.3.1. Bacterial Strains and Growth Conditions

Bacterial strains (*Salmonella enterica* serovar Typhimurium ATCC 14028 and *Escherichia coli* ATCC 25922) were obtained from the American Culture Type Collection (ATCC, Rockville, MD, USA). Bacteria were grown on Mueller–Hinton agar (Sigma Aldrich, St. Louis, MO, USA) at 37 °C for 24 h.

#### 4.3.2. Antimicrobial Susceptibility Tests

The antibacterial activity of prGSE against two Gram-negative bacteria, *S.* Typhimurium and *E. coli*, was determined using the standard microdilution method [40]. Bacterial suspensions were obtained by adjusting bacterial concentration to obtain turbidity visually comparable to the 0.5 McFarland turbidity standard (≈10^8^ CFU/mL). Suspensions were 100-fold diluted with Mueller–Hinton broth (Sigma Aldrich, St. Louis, MO, USA) to obtain a final concentration of 10^6^ CFU/mL, and 100 μL of these suspensions were added to the wells. Serial two-fold dilutions of prGSE in concentrations ranging from 500 μg/mL to 0.97 μg/mL were used to determine Minimum Inhibitory Concentration (MIC). Plates were incubated for 24 h at 37 °C. The MIC endpoint is the lowest concentration of prGSE whereas no visible growth is seen in the wells, compared with the growth control wells. Ciprofloxacin was employed as a control. The tests were conducted twice, in three different replicates.

#### 4.3.3. Anti-Biofilm Activity

The anti-biofilm activity of prGSE was investigated following the method previously described, with some modifications [80]. Here, 10^7^ CFU/mL bacterial suspensions were added to flat-bottomed, 48-well microtiter plates, together with prGSE in concentrations ranging from 250 µg/mL to 15.65 µg/mL, and incubated for 24 h at 37 °C. After that, the supernatant was removed, and bacterial cells and extracellular biofilm matrix were fixed with 200 µL of methanol and incubated at room temperature for 15 min. After removing methanol, the biofilm total biomass was quantified with Crystal Violet (CV; Sigma-Aldrich, Milan, Italy) solution. After the incubation, the biofilm was washed with phosphate-buffered saline (PBS) four times and left to dry. The dye bound to biofilms was solubilized in ethanol 96% for 30 min. The absorbance measurement was recorded at 595 nm using a microplate reader and the total biomass was calculated. Ciprofloxacin was employed as a control. The experiments were conducted in triple, in three different replicates.

#### 4.3.4. In Vivo *G. Mellonella* Survival Assay

In vivo studies were conducted using *G. mellonella* larvae, obtained from Bigserpens (Frosinone, Italy), as previously described [81,82]. *G. mellonella* has been widely accepted as a model organism for studying bacterial infections due to its physiological and immunological similarities with higher organisms, including humans. Using an in vivo model allows researchers to study the actual responses of a living organism to the infection and treatment. Using insect larvae, such as *G. mellonella*, as a model organism is considered more ethically acceptable than using vertebrate animals. It reduces the need for vertebrate animal testing and aligns with the principles of the 3Rs (Replacement, Reduction, Refinement) in animal research. Moreover, *G. mellonella* is relatively easy and cost-effective to maintain in the laboratory. Their short life cycle and ease of handling make them a practical choice for large-scale experiments.

Larvae of *G. mellonella* of 0.3 ± 0.03 g were selected. To prepare the *E. coli* and *S.* Typhimurium inoculum, the strains were cultured in Mueller–Hinton (MH) broth overnight at 37 °C, centrifuged, and suspended in PBS solution. The cell density was determined with an optical density at 600 nm. To confirm the inoculum, the suspension was diluted and placed on MH agar. Colonies were counted after 24 h of incubation at 37 °C. Thirteen groups of larvae (10 larvae for each group, one group for each treatment) were inoculated in the last left proleg with 10^5^ cells of *E. coli* or 10^5^ cells of *S.* Typhimurium together with 10 μL of prGSE at the concentrations of 500 µg/mL, 250 µg/mL and 125 µg/mL. Three groups were defined with, respectively, 500 µg/mL, 250 µg/mL, and 125 µg/mL of prGSE. Three control groups were employed: one group of larvae received no treatment, another group was treated with sterile phosphate-buffered saline (PBS), and two groups were treated with *E. coli* and *S*. Typhimurium, respectively. The larvae were subsequently subjected to incubation at 37 °C and observed for a duration of 120 h. Demise was determined when they exhibited no response to physical stimulation, specifically through the application of slight pressure with forceps. Each experiment was replicated a minimum of three times, and the results were documented as a survival rate percentage.

### 4.4. Statistical Analysis

Data are presented as mean ± standard deviation. Data were analyzed using the GraphPad Prism v.9 (La Jolla, San Diego, CA, USA) software tool. Normally distributed data were analyzed for significance by unpaired *t*-test and two-way ANOVA, followed by post-hoc test (Bonferroni’s). The Mantel–Cox log-rank test was used to judge the statistical significance relative to the control in in vivo tests. Significance was at the 0.05 level.

## Figures and Tables

**Table 1:** Compounds determined by HPLC-DAD, their elution times and concentrations.

Compound	Retention Time (min)	Area	mg/g
Catechin (1)	11.65	408.94	10.92
Procyanidin B2 (2)	16.86	106.99	4.05
Epicatechin (3)	19.03	372	7.66
Epicatechin gallate (4)	30.54	261.12	2.80
Procyanidin Pol 1 (5)	48.59	9694.75	365.48
Procyanidin Pol 2 (6)	52.00	2078.38	78.36

**Table 2:** Molecule amount measured by ^1^H NMR in *V. vinifera* seed extract.

	Molecule	Amount (mg/100 mg Dry Extract)
Amino acids	Leucine	0.01461 ± 0.00073
	Isoleucine	0.01417 ± 0.00071
	Valine	0.0228 ± 0.0011
	Threonine	0.00199 ± 0.00010
	Alanine	0.0403 ± 0.0021
	GABA	0.1199 ± 0.0062
	Glutamine	0.1536 ± 0.0077
	Aspartate	0.372 ± 0.019
	Asparagine	0.027 ± 0.0014
	Phenylalanine	0.034 ± 0.0017
	Tryptophan	0.0838 ± 0.0042
Organic acids	Lactate	0.00633 ± 0.00032
	Acetate	0.056 ± 0.0028
	Citrate	0.1027 ± 0.0051
	Malate	0.445 ± 0.022
	Ascorbate	0.1343 ± 0.0067
	Formate	0.1587 ± 0.0079
Carbohydrates	Glucose	13.55 ± 0.68
	Sucrose	8.17 ± 0.41
Miscellaneous molecules	Ethanol	0.1031 ± 0.0052
	Choline	0.1146 ± 0.0057
	Procyanidin B1	2.66 ± 0.13
	Polymeric Procyanidin (eq. Procyanidin B1)	25.3 ± 1.27
	Trigonelline	0.025 ± 0.0012

**Table 3:** Antibacterial activity of procyanidins-rich grape seed extract against *S.* Typhimurium ATCC 14028 and *E. coli* ATCC 25922 planktonic cells.

	GM MIC_50_ (µg/mL)	GM MIC_90_ (µg/mL)	GM MIC_100_ (µg/mL)
*S.* Typhimurium ATCC 14028	44.17	88.39	222.73
*E. coli* ATCC 25922	55.68	99.21	396.85

MIC_50_ = minimum inhibitory concentration of 50% bacterial growth; MIC_90_ = minimum inhibitory concentration of 90% bacterial growth; MIC_100_ = minimum inhibitory concentration of 100% bacterial growth; GM = geometric mean.

## Data Availability

Data are contained within the article.
